# Nanotechnology Meets Phytotherapy: A Cutting-Edge Approach to Treat Bacterial Infections

**DOI:** 10.3390/ijms26031254

**Published:** 2025-01-31

**Authors:** Katarzyna Pacyga, Paweł Pacyga, Emilia Szuba, Szymon Viscardi, Ewa Topola, Anna Duda-Madej

**Affiliations:** 1 Department of Environment Hygiene and Animal Welfare, Faculty of Biology and Animal Science, Wroclaw University of Environmental and Life Sciences, 50-375 Wroclaw, Poland; 2 Department of Thermodynamics and Renewable Energy Sources, Faculty of Mechanical and Power Engineering, Wrocław University of Science and Technology, 50-370 Wrocław, Poland; pawel.pacyga@pwr.edu.pl; 3 Faculty of Biology and Animal Science, Wroclaw University of Environmental and Life Sciences, 50-375 Wroclaw, Poland; emilia.szuba1@gmail.com; 4 Faculty of Medicine, Wroclaw Medical University, Ludwika Pasteura 1, 50-367 Wrocław, Poland; szymon.viscardi@student.umw.edu.pl (S.V.); ewa.topola@student.umw.edu.pl (E.T.); 5 Department of Microbiology, Faculty of Medicine, Wroclaw Medical University, Chałubińskiego 4, 50-368 Wrocław, Poland; anna.duda-madej@umw.edu.pl

**Keywords:** bioactive compounds, nanoparticles, antibacterial activity, biological activity, drug-resistant bacteria

## Abstract

The increasing prevalence of bacterial infections and the rise in antibiotic resistance have prompted the search for alternative therapeutic strategies. One promising approach involves combining plant-based bioactive substances with nanoparticles, which have demonstrated improved antimicrobial activity compared to their free forms, both in vitro, in vivo, and in clinical studies. This approach not only improves their stability but also enables targeted delivery to bacterial cells, reducing side effects and minimising the risk of resistance development, leading to more effective treatments. This narrative review explores the benefits of combining bioactive plant compounds (berberine, catechin, chelerythrine, cinnamaldehyde, ellagic acid, proanthocyanidin, and sanguinarine) with nanoparticles for the treatment of bacterial infections (caused by *Staphylococcus aureus*, *Enterococcus* spp., *Klebsiella pneumoniae*, *Acinetobacter baumannii*, *Escherichia coli*, *Serratia marcescens*, and *Pseudomonas aeruginosa*), highlighting the potential of this approach to overcome the limitations of traditional antimicrobial therapies. Ultimately, this strategy offers a promising alternative in the fight against resistant bacterial strains, paving the way for the development of more effective and sustainable treatments.

## 1. Introduction

The growing global threat of antibiotic resistance to humans and animals has intensified the search for alternative therapies to treat bacterial infections [1,2]. Antibiotics, once the cornerstone of modern medicine, are becoming less effective due to the rise of multidrug-resistant (MDR) bacteria or “superbugs” (microorganisms that are resistant to most known antimicrobials) [1,3,4]. The probable causes of “global resistance” or antimicrobial resistance (AMR) include excessive or improper antibiotic use in animals (food, pets, aquatic, and farm animals), over-the-counter antibiotic consumption, increased international travel, poor sanitation and hygiene, the release of non-metabolised antibiotics or their residues into the environment through manure or faeces, and inappropriate waste management. These factors redound to the genetic selection pressure for the occurrence and dissemination of MDR bacterial infections [1,3,5]. This alarming trend has prompted the exploration of innovative therapeutic strategies, one of the most promising being the combination of plant-derived bioactive compounds and nanotechnology [6]. Plant-derived compounds, such as alkaloids, flavonoids, terpenoids, and phenolic acids, have long been recognised for their antimicrobial properties [7,8,9]. These natural substances have shown considerable promise in combating various bacterial infections, including those caused by resistant strains [10,11,12]. However, despite their therapeutic potential, the clinical use of plant bioactives is often limited by issues such as low bioavailability, poor water solubility, instability, and rapid metabolism in the body. To overcome these limitations, researchers have turned to nanotechnology, specifically the use of nanoparticles (NPs), to enhance the efficacy of plant bioactive compounds [13].

Nanoparticles offer several advantages in drug delivery, including improved stability, enhanced solubility, and controlled release profiles. The small size of NPs allows for better tissue penetration, higher surface area for interaction, and potential for targeted drug delivery, which is especially important in the treatment of infections in deep or difficult-to-reach tissues [9,14]. Moreover, nanoparticles themselves possess intrinsic antimicrobial properties, which can be leveraged alongside plant bioactives to achieve a synergistic antimicrobial effect [15]. The green synthesis of nanoparticles (NPs) is an environmentally friendly and cost-efficient method that avoids the use of toxic chemicals. This approach has garnered significant interest due to its sustainability, affordability, and excellent stability [8,9].

The combination of plant-based compounds and nanoparticles not only enhances the therapeutic efficacy of the individual components but also helps to overcome the resistance mechanisms employed by bacteria. For example, nanoparticles can disrupt bacterial cell walls, increase the permeability of bacterial membranes, and interfere with biofilm formation, all of which are key factors in bacterial resistance [16]. Moreover, the controlled release of bioactive compounds from nanoparticles can maintain therapeutic concentrations over extended periods, reducing the frequency of dosing and minimising side effects [17]. However, despite the promising results, several challenges remain in the development of nanoparticle-based therapies. These include the optimisation of nanoparticle synthesis, ensuring biocompatibility and safety, and addressing the potential toxicity of nanoparticles, especially in long-term use [6]. Additionally, the regulatory hurdles surrounding the approval of nanoparticle-based formulations for clinical use need to be addressed before widespread application [18].

This literature review presents the findings of studies investigating the effects of plant-derived bioactive compounds (berberine, catechin, chelerythrine, cinnamaldehyde, ellagic acid, proanthocyanidin, and sanguinarine) encapsulated in nanoparticles for the treatment of bacteria colonising wounds, such as *Staphylococcus aureus*, *Enterococcus* spp., *Klebsiella pneumoniae*, *Acinetobacter baumannii*, *Escherichia coli*, *Serratia marcescens*, and *Pseudomonas aeruginosa*. Moreover, in this review, our objective is to clearly distinguish between two types of studies: (1) those focused on the synthesis of nanoparticles (NPs) using plant extracts containing bioactive compounds and (2) those that specifically assess the antimicrobial activity of nanoparticles incorporating only bioactive compounds. It is essential to carefully assess the individual and synergistic antimicrobial effects of AgNPs and any residual bioactive compounds in order to ensure a clear understanding of their respective contributions to the overall antimicrobial efficacy. The presented results suggest that the combination of bioactive compounds and nanotechnology shows considerable promise for addressing bacterial infections, especially those caused by resistant strains. The discussed bioactive compounds are classified into two primary chemical groups. Specifically, berberine, chelerythrine, cinnamaldehyde, and sanguinarine are alkaloids, which are nitrogen-containing compounds primarily derived from plants and renowned for their pharmacological properties. In contrast, catechin, ellagic acid, and proanthocyanidin belong to the polyphenol class, which comprises a wide range of aromatic compounds with phenolic structures commonly found in plants and recognised for their antioxidant effects. The different chemical classes of compounds, such as alkaloids and polyphenols, may exhibit distinct mechanisms of action, which could influence their therapeutic effectiveness when utilised with nanotechnology. This diversity highlights the importance of selecting appropriate bioactive compounds based on their specific properties and potential interactions.

## 2. Insights

In this up-to-date literature review, we provide reports on the antimicrobial activity of nanoparticles prepared using plant bioactive compounds, namely, berberine, catechin, chelerythrine, cinnamaldehyde, ellagic acid, proanthocyanidin, and sanguinarine (Table 1). A summary of the antibacterial properties of these free compounds can be found in our previous work [19]. We focus on bacteria that mainly colonise wounds and can impede wound healing and medical treatment and lead to serious complications, such as *S. aureus*, *Enterococcus* spp., *K. pneumoniae*, *A. baumannii*, *E. coli*, *S. marcescens,* and *P. aeruginosa*.

### 2.1. Berberine (BRB)

#### 2.1.1. In Vitro Assessment of Antimicrobial Activity of Berberine Nanoparticles (BRB-NPs)

**Figure 1:**
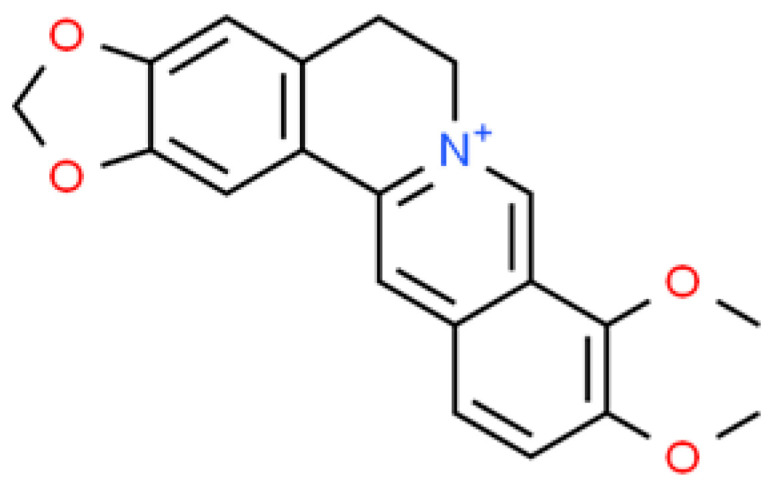
The structural formula of berberine (ChemSpider database).

Based on the available literature, it can be seen that interest in using berberine (the structural formula is shown in Figure 1) for the production of nanoparticles is relatively new. The summary of the antibacterial and biological activity of berberine-based nanoparticles is presented in Table 1. The majority of studies concern *S. aureus*, followed by *E. coli*. One of the earliest reports was published by Yu et al. in 2015 [20]. The authors developed the nanoparticles–beads complex system by the incorporation of berberine-loaded chitosan/fucoidan nanoparticles in the fucoidan-shelled chitosan beads. The nanoparticles–beads complex performed the function of a drug carrier to delay the berberine release in simulated gastric fluid, with an estimated lag time of 2 h. Their results showed that the berberine-loaded beads and nanoparticles–beads complex effectively inhibited the growth of *S. aureus* and *E. coli* and could continuously release the berberine to inhibit bacterial growth for 24 h [20].

In 2019, other authors presented two natural self-assembling modes between berberine (BRB) and two flavonoid glycosides (baicalin (BA) and wogonoside (WOG)) and nanoparticles (NPs) and nanofibers (NFs), which were both mainly governed by electrostatic and hydrophobic interactions. Their study showed that BA, WOG, and BRB first formed one-dimensional complex units and, afterwards, self-assembled into three-dimensional nanostructures. The obtained nanoparticles, with external hydrophilic glucuronic acid, exhibited a stronger affinity to *S. aureus* and *E. coli*, resulting in the collapse of the bacteria population and a decrease in the biofilm [21]. One year later, Hu et al. [22] developed new antibiotic-free berberine/Ag-NPs/silk fibroin (SF)-composited biomimetic calcium phosphate (CaP) scaffolds to enhance the antibacterial and osteogenesis activity. Their results showed that the antimicrobial capacity of BRB coating could be significantly amplified by the introduction of Ag-NPs due to the ability of NPs to penetrate into the pathogen, inactivate metabolic enzymes, and damage the DNA of *S. aureus* [22]. Promising results were also achieved after the use of chitosan nanoparticles (CH-NPs) combined with BRB against *S. aureus*. The inhibitory concentration of CH-NPs (MIC = 150 μg·mL^−1^) combined with berberine (200 μg·mL^−1^) was reduced to 50 μg·mL^−1^ [23]. Li et al. [24] developed a novel packaging film (CC/BRB-B film) by dispersing self-assembled berberine–baicalin nanoparticles (BRB-B-NPs) into a mixed matrix of sodium carboxymethylcellulose-carrageenan (CC). This unique design allowed the use of sunlight to generate reactive oxygen species (ROS), eradicating over 99% of *E. coli* and *S. aureus* within 60 min. Moreover, the film can release BRB-B-NPs to inactivate bacteria under any weather conditions [24]. 

In 2022, the antibacterial activity of pure BRB and BRB-NPs, prepared by antisolvent precipitation (ASP) using glycerol, was evaluated by Nguyen et al. [25]. Their results of the in vitro disk diffusion assay showed greatly reinforced antibacterial activity of BRB-NPs in comparison to pure BRB at the same concentration. The MBC value of berberine NPs against methicillin-resistant *S. aureus* and *E. coli* was found to be 2.0 and 5.0 mg·mL^−1^, respectively [25]. The berberine-bovine serum albumin nanoparticles (BRB-BSA-NPs), prepared by Fatema Younis et al. [26] by employing the desolvation method, were tested against various pathogens, including *E. coli* and *S. aureus*. The authors showed that neither BSA nor BSA-NPs (0.5–40 BSA mg·mL^−1^) exhibited any zones of inhibition. Nevertheless, the encapsulation of berberine (0.01–1.8 BRB mg·mL^−1^) within the core of BSA-NPs considerably increased its antimicrobial effectiveness when compared to pure berberine. The BRB-BSA-NPs increased the inhibition zone diameters and decreased the minimum inhibitory concentrations in comparison to BRB; for instance, *E. coli* and *S. aureus* both illustrated an inhibition zone of 32.7 ± 1.3 vs. 29.3 ± 1.5 mm and an MIC value of 6.1 ± 0.2 vs. 8.2 ± 0.2 μg·mL^−1^, while for *S. aureus* it was 32.7 ± 1.3 vs. 30.8 ± 1.7 mm and an MIC value of 3.2 ± 0.1 vs. 10.8 ± 0.4 μg·mL^−1^ [26]. Another study demonstrated the enhanced antibacterial effects of BRB-NPs, formed by antisolvent precipitation, compared to pure BRB. The minimum bactericidal concentration of BRB-NPs against methicillin-resistant *S. aureus* and *E. coli* amounted to 2.0 and 5.0 mg·mL^−1^, respectively. Moreover, the transmission electron microscopy allowed the observation that BRB-NPs surrounded and severely damaged the bacterial cells [25]. The impact of biodegradable and antibacterial packaging films, prepared by incorporating self-assembled berberine-cinnamic acid nanoparticles (BC-NPs) into the film’s matrices, exhibited strong antibacterial ability against both *E. coli* and *S. aureus*. The measurement of their fluorescence spectrum and ROS production confirmed their enhanced antimicrobial activity under white light [27]. Sitah Alharthi et al. [28] proposed the development of synergistic therapies to treat superbug infections through the encapsulation of berberine chloride (BRB) (sortase A inhibitors; SrtAIs) into MCM-41 mesoporous silica nanoparticles (MSNPs) or a phosphonate-modified analogue (MCM-41-PO_3_^−^). Their results showed that the MCM-41 and MCM-41-PO_3_^−^ formulations significantly improved the aqueous solubility of BRB. Moreover, the MIC values for MCM-41-PO_3_^−^ formulations were lower compared to the SrtAI/MCM-41 formulations against the tested methicillin-sensitive and methicillin-resistant *S. aureus*, *E. coli* (with the exception of the cases of BR/MCM-41 *P. aeruginosa*) [28].

Further studies on co-assembled berberine–tannic acid nanoparticles (BRB-TA-NPs) [29] and berberine and epigallocatechin-3-gallate nanoparticles (BRB-EGCG-NPs) [30] were published in 2023 by Zheng and co-workers. The authors presented that BRB-TA-NPs, obtained via solvent evaporation, exhibited the best antimicrobial activity against *S. aureus* and multidrug-resistant *S. aureus* in relation to the activities of individual bioactive compounds as well as tested antibiotics (benzylpenicillin potassium and ciprofloxacin). Even at low concentrations (15.63 μg·mL^−1^), the bacteria inhibition rate of BRB-TA-NPs was over 80%. This efficacy was achieved by adhering to microbial cell walls, causing membrane damage and ATP leakage [29]. Similarly, the BRB-EGCG-NPs showed increased biocompatibility and antibacterial activity relative to free BRB and the aforementioned first-line antibiotics. The authors also presented the synergistic bactericidal effect of the combination of BRB and EGCG. Particularly noteworthy is also the finding that BRB-EGCG-NPs exhibited no toxic effects on the major organs of mice [30]. The study conducted by Alharthi et al. [31] aimed at developing β-lactoglobulin protein nanoparticles (P-NPs) for the encapsulation of berberine. Their activity, alone and combined with the pexiganan, indolicidin, and mastoparan derivative, was tested against methicillin-sensitive and methicillin-resistant *S. aureus* as well as *E. coli* and *P. aeruginosa*. After the treatment, the growth of Gram-positive (MIC: 125 μg·mL^−1^) and Gram-negative (MIC: 250–500 μg·mL^−1^) bacteria were inhibited. The BRB-P-NPs with pexiganan or indolicidin demonstrated synergy against *S. aureus* [31]. 

The most recent studies demonstrated that the berberine and chlorogenic acid-assembled nanoparticles (BRB-CGA-NPs) were effective in combating MRSA through the destruction of bacterial cell wall structures and membranes, influencing the stability of the bacterial structure [32]. Andima et al. [33] evaluated the impact of lactoferrin nanoparticles (Lf-NPs) loaded with a dual drug combination of berberine or sanguinarine with vancomycin or imipenem against *S. aureus*. The authors observed that Lf-NPs loaded with combinations of berberine and imipenem (Lf-BRB-I) and sanguinarine and vancomycin (Lf-SAN-V) caused total eradication of intracellular *S. aureus* at 50 μg·mL^−1^ [33]. Abd El-Hamid et al. [4] developed novel mesoporous silica nanoparticles (MPS-NPs) for delivering free berberine (Free-BRB) and assessed their in vitro activity against robust biofilm-forming and multi-virulent vancomycin-resistant *S. aureus* (VRSA). The obtained formulation exhibited strong in vitro antimicrobial, antibiofilm, anti-quorum sensing, and antivirulence activity at their sub-inhibitory concentrations (SICs) against VRSA. Similarly, MRSA growth and biofilm formation were entirely inhibited by berberine at sub-MIC doses [4]. The effectiveness of BRB-Au-NPs against MRSA was also proven by Sadeghi et al. [34]. The in vitro antibacterial tests showed that the conjugated BRB-Au-NPs exhibited a lower MIC value against MRSA (109.5 μg·mL^−1^) than free BRB (165 μg·mL^−1^). The application of free BRB and conjugated BRB resulted in 13.9 and 22.33% biofilm eradication, respectively. Moreover, the cytotoxicity study on the mouse fibroblast cell line proved the biosafety of free as well as conjugated BRB at their MIC concentration (almost 100% cell viability after exposure) [34]. It was also shown that the release of berberine hydrochloride encapsulated in mesoporous titanium nanoparticles (MT-NPs), modified with functionalised UV-responsive ethylene imine polymer (PEI), can be controlled by UV irradiation and the bacterial lethality of up to 37.8%, can be achieved after only 8 min of irradiation. The MIC value of BRB-MT-NPs-PEI amounted to 1 mg·mL^−1^ against *E. coli* (the bacterial growth was entirely inhibited during 24 h, and the MBC value amounted to 5 mg·mL^−1^ [35]. In another study, the berberine-loaded zein nanoparticles (BRB-Z-NPs), obtained by electrospray, exhibited high inhibition of *E. coli* growth (−29% and −46% for empty and berberine-loaded particles, respectively) compared to the medical-grade metal substrates [36]. Jingjing Zhang et al. [37] developed the composite nanoagent (B-E-M-G-NPs) by integrating berberine (BRB) and epigallocatechin gallate (EGCG), coating with manganese dioxide nanoshells (MnO_2_-NSs), and glucose oxidase (GOX). It was found that the B-E-M-G-NPs, which acted as photosensitisers, effectively produced reactive oxygen species (ROS), which enhanced the eradication of bacteria with the assistance of O_2_. Due to the synergistic function of the cascaded reaction, the composite nanoagent showed high antibacterial efficacy even for multidrug-resistant (MDR) bacteria of methicillin-resistant *S. aureus* and kanamycin-resistant *E. coli* (KREC). In the absence of light, the antibacterial efficacy of B-E-M-G-NPs was limited for both MRSA and KREC. However, under light irradiation, the antibacterial efficacy reached 60.4% against MRSA and 74.8% against KREC. The antibacterial efficacy was significantly improved (to 100%) for both bacteria strains under the synergistic effect of light irradiation and glucose (more ROS was generated with the assistance of generated O_2_) [37]. Fu et al. [38] demonstrated that the nanodrug graphene oxide (GO) assembled with berberine and aloe-emodin (AE) could significantly enhance antibacterial activity against *S. aureus* through direct interactions with bacterial cells, leading to the downregulation of bacterial nutrient metabolism, inhibition of biofilm formation, and suppression of toxin-related gene expression. This formulation exhibits the ability to effectively reduce *S. aureus* populations in mouse mammary tissue, mitigate bacterial-induced damage, and alleviate inflammation symptoms [38]. Moreover, berberine-loaded polymeric nanoparticles exhibited properties that effectively reduced the viability of *Enterococcus faecalis* in biofilms, which are typically resistant to conventional treatments. The population of *E. faecalis* was susceptible to berberine-loaded poly lactic-co-glycolic acid (PLGA) nanoparticles. The reduced viability of the sessile endodontic bacteria was observed at 30 and 40 μg·mL^−1^ concentrations of BRB-loaded PLGA-NPs upon 24 h of exposure [39].

#### 2.1.2. In Vivo Assessment of Antimicrobial Activity of Berberine Nanoparticles (BRB-NPs)

Various types of BRB-loaded NPs demonstrate superior antimicrobial activity compared to pure BRB in in vivo studies. The study conducted on a rat model confirmed the high encapsulation efficiency, prolonged release of BRB, and enhanced biological stability using hybrid polymer-lipid nanoparticles (PEG-lipid-PLGA NPs). BRB administered in this form was significantly better absorbed in the gut, with its bioavailability markedly increased compared to BRB alone [40]. The effectiveness of BRB-Au-NPs against MRSA was demonstrated by Sadeghi et al. [34]. The in vivo study with the use of an infected skin model showed an MRSA survival rate of 2.7% and 26% in groups treated with conjugated and free BRB [34]. In another study conducted by Hongyu Li et al. [41], the effectiveness of the complex of fructan-based nanoparticles and berberine was proved. The obtained nanoparticles significantly ameliorated the inflammatory response of sodium dextran sulphate (DSS)-induced colitis in mice by inhibiting the activation of the NF-κB/STAT-3 pathway and enhancing tight junction protein expression in vivo. This complex also deteriorated the increased richness of detrimental flora in colitis, for example, *Enterobacteriaceae* such as *Escherichia* and *Shigella* [41].

#### 2.1.3. Antimicrobial Activity of Nanoparticles Based on Plant Extracts Containing Berberine

The effect of synthesised silver nanoparticles (Ag-NPs) using berberine plant extract against multidrug-resistant *A. baumannii* and *P. aeruginosa* was evaluated by Maedeh Tahan et al. [42]. Their experiments showed that BRB-Ag-NPs inhibited the growth of bacteria at lower concentrations than antibiotics, and the bacterial strains could not grow even at low concentrations of BRB-Ag-NPs [42].

**Table 1:** The antibacterial mechanisms of berberine-based nanoparticles.

The Antibacterial Mechanisms	Reference
adherence/internalisation to bacteria cell walls; membrane damage and ATP leakage; inactivation of metabolic enzymes	[21,22,23,25,29,34,39]
inhibition of bacteria adherence and proliferation; inhibition of bacterial biofilm formation, thereby enhancing antimicrobial, anti-biofilm, anti-quorum sensing, and anti-virulence effects	[20,36]
ROS generation	[23]
regulation of relative homeostasis; inhibition of the key intracellular mechanism	[41]

There are no reports in the available literature regarding the use of berberine-based nanoparticles in the treatment of infections caused by *K. pneumoniae* and *S. marcescens*, and future research should focus on exploring these analyses in greater depth.

### 2.2. Catechin (CT)

#### 2.2.1. In Vitro Assessment of Antimicrobial Activity of Catechin Nanoparticles (CT-NPs)

**Figure 2:**
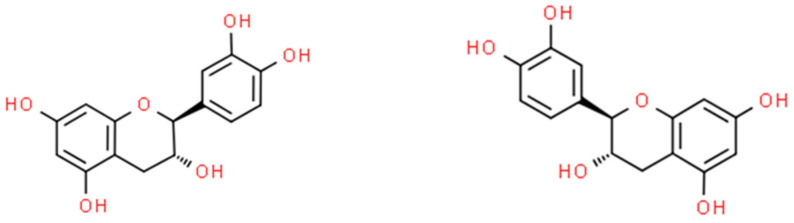
The structural formula of L-(−)-catechin (on the **left**) and D-(+)-catechin (on the **right**) (ChemSpider database).

The interest in nanoparticles based on catechin (Figure 2) started in 2015 when Huanhuan Li et al. [43] developed a simple and low-energy-consuming approach to synthesise catechin-based Cu-NPs (CT-Cu-NPs). Their results showed that treatment with the concentrations of 10 mg·L^−1^ and 20 mg·L^−1^ effectively inhibited *S. aureus* and *E. coli* growth up to 90 and 85% within 3 h, respectively. The application of 20 mg·L^−1^ and 40 mg·L^−1^ CT-Cu-NPs caused the full eradication of *E. coli* and *S. aureus* within 3 h [43]. One year later, Hongcai Zhang et al. [44] published their findings on the antibacterial activity of catechin or CT-Zn complex-loaded β-chitosan nanoparticles (β-CH-NPs), obtained by ionic gelation technology, against *E. coli*. The MIC and MBC values of CAT-Zn complex loaded β-CS NPs were 0.063 and 0.125 mg·L^−1^, respectively. Moreover, the encapsulation of the CT-Zn complex in β-CS NPs improved the antibacterial activity of CT and the CT-Zn complex [44].

Two years later, the ability of chitosan-based nanoparticles loaded with catechin and quercetin (also obtained through the ionic gelatine reaction) to inhibit the growth of *S. aureus* and *E. coli* was also confirmed by other authors [45]. Susan Oliver et al. [46] explored two techniques aimed at improving the stabilising capacity of catechin, namely, cross-linking CT with sodium tetraborate (borax) and preparing water-soluble oligomer from catechin (polycat). It was found that polycat-Ag-NPs showed superior antimicrobial activity, exhibiting the MIC value of only 1.25 μg·mL^−1^ (Ag) for *P. aeruginosa* and *A. baumannii*. Additionally, polycat-Ag-NPs also demonstrated enhanced antibiofilm activity; namely, the concentration of 5 μg·mL^−1^ (Ag) resulted in a 99.9% reduction in biofilm cell viability and a 99.1% reduction in biofilm biomass with polycat-Ag-NPs [46].

The antimicrobial activity of catechin and rare-earth ions (Re^3+^) nanoparticles (Re-CT-NPs) coated on a polyamide (PA) membrane was tested against *P. aeruginosa* under both static and dynamic conditions. The Re-CT nanocoating significantly inhibited bacterial growth (over 90%) and attachment and demonstrated excellent reusability and long-term stability [47]. The application of catechin-functionalised ZnO nanoclusters, synthesised via a hydrothermal method, resulted in a high inhibition ratio for *S. aureus* and *E. coli*. The MIC and MBC values of CT-ZnO against *E. coli* and S. *aureus* were the same and amounted to 29.8 and 69.5 μg·mL^−1^, while the values for pure CT were 919 and 1838 μg·mL^−1^ for *E. coli* and 460 and 919 μg·mL^−1^ for *S. aureus* [48]. 

The catechin-based titanium oxide–gold nanocomposites (CT-TiOx–Au-NCs) were highly efficient for the photoinactivation of *E. coli* and methicillin-resistant *S. aureus* through the photodynamic generation of reactive oxygen species (ROS) and damage to the bacterial membrane [49]. Ivanova et al. [50] explored the synergistic activity of chlorhexidine–silver nanoparticles (Cx-Ag-NPs), obtained with GT-extract, against *S. aureus* and *E. coli*. The conjugates presented potentiation in their effects against *S. aureus* and synergism against *E. coli* with MIC values of SN at 5.5 μg·mL^−1^ + Cx 8.8 μg·mL^−1^ [50].

#### 2.2.2. In Vivo Assessment of Antimicrobial Activity of Catechin Nanoparticles (CT-NPs)

The literature on the effect of CT-charged nanoparticles (NPs) on CT activity is limited and clearly warrants further investigation by researchers. Nevertheless, this issue has already been addressed by Hsieh et al. [51]. Their studies demonstrated that the transfersome form of catechin exhibits significantly better skin penetration than the CT solution. The transdermal delivery of catechin via transfersomes was found to achieve 85% skin penetration, compared to 78% with the pure CT solution. Furthermore, transfersomes were shown to be more effective in inhibiting tyrosinase. Conversely, animal studies using guinea pigs revealed that this form of CT delivery improved skin biocompatibility when applied topically. The animals’ favourable tolerance suggests that transfersomes could serve as a promising strategy for treating oxidative skin damage resulting from UV exposure [51,52].

#### 2.2.3. Antimicrobial Activity of Nanoparticles Based on Plant Extracts Containing Catechin

The use of *Azadirachta indica* (neem) leaf extract, containing 0.532 mg·g^−1^ of catechin, can be successfully used for the production of Fe-NPs with the antimicrobial activity against *E. coli*, *S. aureus*, and *P. aeruginosa* [53]. 

Another study employed green tea (GT) extract (a source of catechins) to produce Ag-NPs as well as Ag-NPs coated with polyethylene glycol (PEG) to enhance their dispersion and biocompatibility. The MIC and MBC values obtained for the Ag-NPs after 24 h of bacterial incubation were the same and amounted to 250 μg·mL^−1^ for *S. aureus* and *K. pneumoniae*, 15 μg·mL^−1^ for *E. soli*, and 30 μg·mL^−1^ for *P. aeruginosa*. In the case of the Ag-NPs-PEG, the values were as follows: 250 μg·mL^−1^ for *S. aureus*, 500 μg·mL^−1^ for *K. pneumoniae*, 60 μg·mL^−1^ for *E. coli*, and 125 μg·mL^−1^ for *P. aeruginosa*. It was also suggested that Ag-NPs can attach to the bacterial cell membrane, mainly through interactions with proteins containing sulphur residue. This action disturbs membrane permeability, which then causes leakage of intracellular content and compromises respiration functions of the cell, leading to DNA damage, production of reactive oxygen species and cell death [54].

Additionally, it was found that the extract of *Acca sellowiana* fruit can also constitute a source of catechin and can be used for the production of Ag-NPs and zero-valent iron (ZVFe) nanoparticles to inhibit the growth of *P. aeruginosa*, *K. pneumoniae*, *S. aureus*, *E. faecalis*, *A. baumannii*, and *E. coli* [55]. 

The zone of *E. coli* inhibition after the treatment with silver nanoparticles (50 μg·mL^−1^) based on green tea extract, EGCG, and CT were 28.5, 26.5, and 28.0 mm, respectively, while for *S. aureus* they were 20.5, 19.0, and 21.0 mm, respectively. These NPs showed enhanced antibacterial activity in comparison to blank samples: free GT extract, EGCG, and CT [56]. 

*Cotoneaster* extract-based copper oxide nanoparticles (CuO-NPs), which contain 122.46 μg of catechin equivalent per mg nanoparticle, represent significant antimicrobial activity against *E. coli* and *S. aureus* with an inhibition zone of 19 ± 1 mm and 23 ± 2 mm, respectively, employing disk diffusion [57]. Alowaiesh et al. [58] utilised the agro-industrial residues, namely, olive leaf waste (OL), as the source of phenolic compounds (among others, catechin 720.39 μg·mL^−1^) for the production of Ag-NPs. The antibacterial properties, assessed by measuring the inhibition zone diameter, showed that the OL-Ag-NPs, at concentrations ranging from 2.5 to 20 μg·mL^−1^, significantly inhibited the growth of multidrug-resistant (MDR) bacteria, including *S. aureus* and *E. coli* [58]. Table 2 provides an overview of the mechanisms of action of catechin nanoparticles.

Future research should concentrate on assessing the effects of catechin-based nanoparticles on *S. marcescens*.

### 2.3. Chelerythrine (CHE)

#### 2.3.1. In Vitro Assessment of Antimicrobial Activity of Chelerythrine Nanoparticles (CHE-NPs)

To combat infections caused by ESKAPE pathogens (*K. pneumoniae*, *A. baumannii*, *P. aeruginosa*, *Enterobacter* spp., and *E. coli*), LNP-CHE-CST@hydrogel nanoparticles were synthesised by Wei et al. [61]. This hydrogel system contained liposomal chelerythrine and colistin, which target ROS and the FtsZ protein. Bacterial cell death was induced through DNA fragmentation, caspase accumulation, membrane depolarisation, exposure to phosphatidylserine, and chromosome condensation [61].

**Figure 3:**
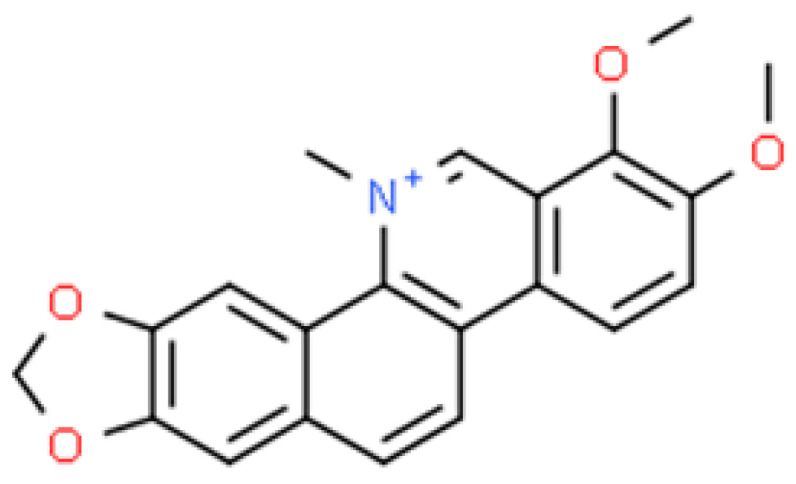
The structural formula of chelerythrine (ChemSpider database).

The structural formula of this bioactive compound is shown in Figure 3.

#### 2.3.2. In Vivo Assessment of Antimicrobial Activity of Chelerythrine Nanoparticles (CHE-NPs)

Furthermore, the antibacterial LNP-CHE-CST@hydrogel nanoparticles obtained by Wei et al. [61] were shown to accelerate the healing of skin infections in mice. Following just two doses of LNP-CHE-CST@hydrogel nanoparticles (20 mg·cm^−2^), bacterial levels in the skin were nearly eradicated within 24 h, as evidenced by fluorescence tracking. Given its strong efficacy and excellent safety profile, this hydrogel shows considerable potential for clinical translation in the treatment of Gram-negative pathogen infections [61].

#### 2.3.3. Antimicrobial Activity of Nanoparticles Based on Plant Extracts Containing Chelerythrine

For the first time, silver nanoparticles were eco-synthesised using an aqueous extract of *Chelidonium majus* L. The resulting silver-based biohybrids demonstrated potent antimicrobial activity against *E. coli* (with an inhibition zone measuring 51 mm in diameter) and showed improved physical stability compared to phyto-nanoAg alone [62]. In the work of Dobrucka et al. [63], the extract of *Chelidonium majus* was used for the biological synthesis of ZnO nanoparticles. The obtained nanoparticles were effective bioactive factors that exhibited a biocidal effect against *S. aureus*, *P. aeruginosa*, and *E. coli*. The MIC (20 µM) and MBC (40 µM) values for the standard strain *S. aureus* were comparable to those obtained for amikacin (25 µM and 25 µM, respectively), which was used as a reference antibacterial agent. The ZnO nanoparticles were slightly less active against clinical strains of *S. aureus* (MIC value of 40 µM and MBC value of 120 µM) and *E. coli* (both MIC and MBC values of 80 µM). The strains of *P. aeruginosa* showed the lowest sensitivity to the synthesised ZnO nanoparticles, but the MIC (120 µM) and MBC (640 µM) values were still relatively low. The authors indicated that the synthesised ZnO nanoparticles have the potential to be developed as antimicrobial agents effective against a broad spectrum of microorganisms, helping to control and prevent the further spread of microbes [63]. Table 3 presents a summary of the mechanisms through which chelerythrine nanoparticles exert their effects.

### 2.4. Cinnamaldehyde (CA)

#### 2.4.1. In Vitro Assessment of Antimicrobial Activity of Cinnamaldehyde Nanoparticles (CA-NPs)

**Figure 4:**
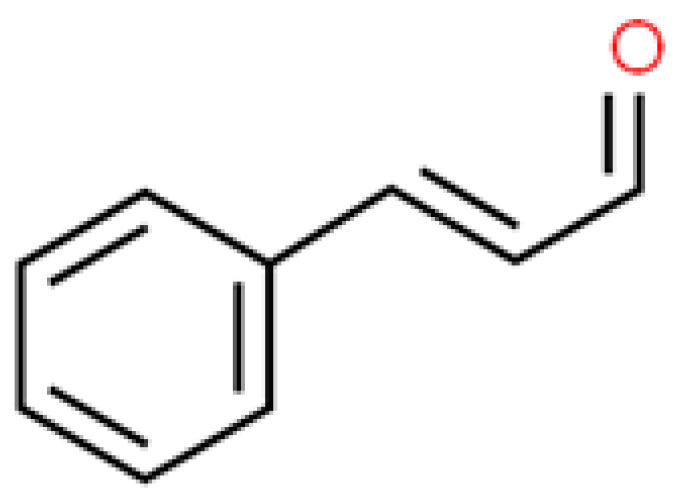
The structural formula of cinnamaldehyde (ChemSpider database).

The treatment of *E. coli* in planktonic form with cinnamaldehyde (the structural formula is presented in Figure 4) packaged into mesoporous silica nanoparticles resulted in enhanced antimicrobial activity. Moreover, these nanoparticles exhibited activity against *P. aeruginosa* biofilms that have inherent resistance to antimicrobial agents [64]. Antibiofilm properties of Au-NPs synthesised with CA (the concentration ranged from 0 to 0.01%) were investigated against *P. aeruginosa*, *E. coli*, *S. aureus*: MSSA (meticillin-sensitive) and MRSA (methicillin resistant). More than a 90% reduction in *E. coli* and MRSA biofilm-forming ability was observed. Other bacteria were also strongly inhibited in terms of biofilm formation, namely MSSA by 85% and *P. aeruginosa* by 65% [65]. In a similar study of the same authors, the above-mentioned compound was used at an even lower concentration (0.005%) against the same pathogens. In the SEM/TEM analysis, the thickening of bacterial cell walls, premature cell division, disintegration in the cytosol, and finally, cell lysis were observed [66]. The decahedral cinnamon nanoparticles in pure honey obtained via pulse laser ablation in liquid (PLAL) technique at varied laser ablation energy (LAE, 0–180 mJ) were evaluated for their bactericidal activity against *E. coli* using agar well diffusion and optical density analyses. The authors found that the prepared nanoparticles exhibited promising bactericidal activity [67]. The application of cinnamaldehyde-encapsulated chitosan nanoparticles (CA-NPs) altered the swimming and swarming motility of *P. aeruginosa*. Additionally, in vitro studies confirmed the slow and sustained release of CA [68]. 

The chitosan NPs cross-linked with cinnamaldehyde, used in the study conducted by Gadkari et al. [69], exhibited antimicrobial activity against *S. aureus* and *E. coli* (the MBC and MIC values amounted to 10 and 5 mg·mL^−1^, respectively, both for *E. coli* and *S. aureus*. Moreover, the concentration of 1 mg·mL^−1^ showed a 47% reduction in density for *S. aureus* and 46% for *E. coli* [69]. To deliver *trans*-CA to bacterial cells, a combination of poly(D,L-lactide-co-glycolide; PLGA) and chitosan (CH) was developed by Pola et al. [70]. The MIC value of the encapsulated CA was lower (32 μg·mL^−1^) than that of the free CA (512 μg·mL^−1^) for *S. aureus*. Moreover, the addition of chitosan presented an even lower MIC value (16 μg·mL^−1^). The free CA showed a bactericidal effect against tested bacteria at 1024 µg·mL^−1^, while the combination of CA and CH amounted to 64 µg·mL^−1^ [70].

Gadkari et al. [71] synthesised the chitosan-cinnamaldehyde cross-linked nanoparticles (CH-CA-NPs) via a green route and deposited, in alternating combination with sodium tripolyphosphate (TPP), over a polyester nonwoven fabric using a layer-by-layer coating technique. It was found that the 10.5 bi-layers of CH-CA-TPP-NPs, even at low concentrations of NPs (0.1 *w*/*v*), showed approximately 98% and 99% antibacterial activity against *S. aureus* and *E. coli* bacteria, respectively. Moreover, a significant reduction in the number of bacterial colonies as compared with untreated fabric was observed [71]. Chotchindakun et al. [72] used polyhydroxybutyrate-co-hydroxyvalerate (PHBV), which is considered a suitable polymer for drug delivery systems and bone tissue engineering due to its biocompatibility and biodegradability. The authors incorporated mesoporous bioactive glass nanoparticles (MBG-NPs) into PHBV to enhance its bioactivity and CA (5, 10, and 20%) to implement antibacterial activity. The augmented antimicrobial activity was observed with increasing CA concentration. The NPs with 20% of CA presented the lowest relative bacterial viability at 17.6% within 24 h for *S. aureus* and at 17.0% within 3 h for *E. coli*. Additionally, the release of CA was observed for up to 7 days [72]. 

The investigation conducted by Xu et al. [73] revealed the promising effectiveness of CH-CA-NPs on *S. aureus* biofilm eradication (up to 61%). The MIC value for the tested NPs was equal to 2.5 mg·mL^−1^. Based on the results, the authors suggest the possibility of using CA-based NPs in, for example, food preservation [73]. The positively charged multifunctional nanoplatforms of SiO_2_-CA-CuS nanospheres were able to adhere to the negatively charged surface of bacteria and cause their rapid eradication through the synergistic action of the released CA and heat produced under near-infrared light (NIR) irradiation at 980 nm. The sterilisation efficiencies for *E. coli* and *S. aureus* reached 99.86% and 99.84%, respectively. Moreover, they achieved high biocompatibility and effectiveness in accelerating *S. aureus*-infected wound healing at a low photothermal temperature (45 °C) [74]. The application of CH-CA-NPs, embedded into chitosan/poly(vinyl alcohol)/fish gelatin (CPF) ternary matrices, against food-borne pathogens, such as *S. aureus* and *E. coli*, was also demonstrated by Hosseini et al. [75]. The analysis of storage quality indices showed that the shelf-life of rainbow trout fillets wrapped in CPF-CH-CA-NPs 0.25 was extended to 12 days [75]. The activity of encapsulated CA-loaded CH-NPs into guar gum/poly(methylvinylether-alt-maleicacid) (GG/PMVE-MA) hydrogels was tested against *E. coli* and *S. aureus*. The zone of inhibition (ZOI) observed for Gram-positive bacteria was 14.3 ± 1.12 mm. However, due to the more complex structure of the Gram-negative microbial cell wall, the penetration of NPs with CA was prevented [76]. 

The effect of biogenic (Bio)-CA-Ag-NPs on *S. aureus* and *E. coli* and for sanitation activities on fresh sweet grape tomatoes were evaluated by Batista et al. [77]. The application of BioAgNP inhibited the growth of the tested bacteria, while in the case of the sanitisation of fruits, CA (156 µg·mL^−1^) combined with Bio-Ag-NPs (31.25 μM) at subinhibitory concentrations inhibited the growth of *E. coli* after only 5 min of contact. The exposed samples showed no growth of *E. coli* during their shelf life [77]. The two-tailed antimicrobial amphiphiles (T^2^A^2^) composed of nitric oxide (NO)-donor (diethylenetriamine NONOate, DN) and various aldehydes, among others, cinnamaldehyde were investigated by Hu et al. [78]. The results showed that the CA-T^2^A^2^ assemblies exhibited excellent bactericidal efficacy; they killed multidrug-resistant *S. aureus* and eradicated their biofilms via multiple mechanisms. Moreover, CA-T^2^A^2^ assemblies rapidly eradicated bacteria and alleviated inflammation in the subsequent murine infection models. These combinations were capable of secreting nitric oxide as well as increasing the permeability of the microbial cell membrane. Furthermore, it was confirmed that preparation led to the inhibition of enzymes that produce ATP, which is necessary for many cellular processes [78]. 

A nanodevice proposed by Morellá-Aucejo et al. [79], consisting of mesoporous silica nanoparticles loaded with cinnamaldehyde and functionalised with the polypeptide ε-poly-L-lysine, was tested against *E. coli* and *S. aureus*. An increase in the delivery of CA through the biocontrolled uncapping mechanism triggered by proteolytic enzymes was observed. After the treatment with the nanodevice, the antimicrobial efficacy of CA was enhanced in comparison to the free CA, namely, 52-fold for *E. coli* and 60-fold for *S. aureus*, which can be assigned to the diminishment of its volatility due to the encapsulation in the silica matrix and thus the increase in its local concentration [79].

#### 2.4.2. In Vivo Assessment of Antimicrobial Activity of Cinnamaldehyde Nanoparticles (CA-NPs)

The synthesised cinnamaldehyde-based sulfonated all-polyester prodrug (CA-SAPP) nanoparticles demonstrated antibacterial activity against *E. coli* and *S. aureus*, including methicillin-resistant *S. aureus*. These nanoparticles effectively bound to, aggregated, and precipitated the bacteria. In vivo studies further revealed that CA-SAPP nanoparticles promoted the healing of *S. aureus*-infected wounds with minimal systemic toxicity and high biocompatibility [80].

#### 2.4.3. Antimicrobial Activity of Nanoparticles Based on Plant Extracts Containing Cinnamaldehyde

Marcondes et al. [81] evaluated the effect of synthesised poly(vinyl alcohol)/poly(glycerol) dendrimer hydrogel incorporated with silver nanoparticles (PVA/PGLD-Ag-NPs) using *Cinnamomum verum* extract containing cinnamaldehyde. The inhibition zone for *E. coli*, investigated by disk diffusion method, ranged from 6.05 ± 0.03 to 7.93 ± 0.05 mm (depending on the concentration) [81].

The polyvinyl alcohol (PVA) films combined with nanoparticles and cinnamon essential oil (CEO) were proposed as an alternative to the currently used food packaging materials. The PVA-based nanocomposite films CEO-ZnO-NPs and nanocellulose (NC) were synthesised. It was found that the presence of ZnO-NPs increased the release of cinnamaldehyde from 31.16 to 44.23% and further enhanced to 71.82% when nanocellulose was used. The incorporation of the NPs also improved the hydrodynamic and mechanical properties of the films. The authors also observed the reduced levels of hypoxanthine and methylmalonic acid and increased level of proline in the case of *S. aureus*, which bacteria produced as a result of oxidative stress caused by the use of cinnamon essential oil [82]. The synthesised Ag-NPs, by means of the one-pot green approach and cinnamon bark extract (containing a cinnamaldehyde as the main component), demonstrated promising potential as an antimicrobial agent against *E. coli*, *K. pneumoniae*, *P. aeruginosa*, and *S. aureus* due to enhanced surface stability [83].

The antimicrobial activity of the cinnamaldehyde-based nanoparticles is summarised in Table 4.

There are no reports in the available literature regarding the impact of cinnamaldehyde-based nanoparticles on *Enterococcus* spp., *A. baumannii*, and *S. marcescens*, and further studies could explore their potential antimicrobial effects, mechanisms of action, and therapeutic applications.

### 2.5. Ellagic Acid (EA)

#### 2.5.1. In Vitro Assessment of Antimicrobial Activity of Ellagic Acid Nanoparticles (EA-NPs)

**Figure 5:**
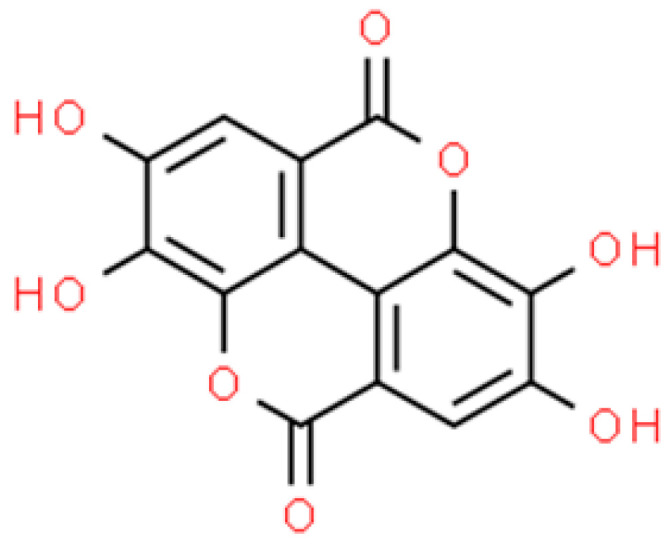
The structural formula of ellagic acid (ChemSpider database).

The activity of zein-based NPs loaded with EA (the structural formula is shown in Figure 5) (EA-Z-NPs) against bacterial pathogens, including *S. aureus*, was also assessed. The microdilution method showed the MIC and MBC values for the nanoparticles were 72 and 144 μg·mL^−1^, respectively. Furthermore, they were characterised by their ability to eradicate the *Staphylococcus* biofilm, resulting in a 60% decrease in bacteria viability. Promising results were also noted against *P. aeruginosa*. The EA-Z-NPs showed an MIC of 36 μg·mL^−1^ and an MBC value of 72 μg·mL^−1^. In turn, the eradication activity against mature bacterial biofilm was lower than in the case of *S. aureus*, and the reduction of the biofilm viability by about 30% was observed [84]. 

El-Sonbaty et al. [85] also evaluated the antimicrobial activity, among others, against *S. aureus* of newly synthesised gallium nanoparticles coated with ellagic acid (EA-G-NPs). In the Kirby–Bauer test, the application of 1 mg·mL^−1^ of EA-G-NPs per disc resulted in the zone of inhibition of 14 mm, while in the control group treated with gentamicin at the concentration of 4 pg·mL^−1^, the observed ZOI was 24 mm. The EA-G-NPs were also relatively active against *E. coli*, where the value of the ZOI was 12 mm, while in the gentamycin group, it was 30 mm [85]. Tavares et al. [84] evaluated the antibacterial activity of the chitosan/zeine/gelatin film loaded with EA nanoparticles. In the in vitro test, in the group with the prepared film, a decrease in the number of *S. aureus* cells by 2 logarithmic units was observed [84]. The activity of EA in the form of lipid NPs (liposomes) was also examined in relation to MDR *A. baumannii*. The MIC value amounted to 32 μg·mL^−1^, while the MBC value to 64 μg·mL^−1^. In turn, the zone of inhibition was concentration-dependent and ranged from 11 to 28 mm. In addition, the nanoparticles in the MBC concentration inhibited the formation of biofilm of *A. baumannii* to 13% of the initial state [86]. 

Wang et al. [87] investigated the antimicrobial properties of Au-NPs modified with EA against the ESKAPE group pathogens. In the case of *S. aureus*, the MIC and MBC values were equal to 3.12 μg·mL^−1^ (for 3 tested strains: Xen36, 6114, and ATCC 12600). The SEM study showed that exposure to this concentration led to cell contraction and induced direct cell wall damage. An increased ROS accumulation was also found inside the tested cells. For the Xen36 strain, in vitro studies were also conducted on the ability of the NPs to eradicate the pathogen biofilm. At the concentration of 6.25 μg·mL^−1^, damage to almost 95% of the biofilm was observed. Moreover, a decrease in the biofilm biomass by 57% was demonstrated with simultaneous determination of a decrease in the number of bacteria in the biofilm by 5 logarithmic units. The molecular basis of the NPs’ activity was assessed by the analysis of *S. aureus* transcriptome. It showed an increase in the expression of several genes (*ABC.PE.A*, *ABC.PE.P1*, *ABC.PE.S*, and *dltD*) and reduced of others (*scin*, *efb*, *ribD*, *sspA*, *hlg*, *etc*, *hld*, *ribD*, *fadA*, and urea). Importantly, the diminished expression concerned mainly genes related to purine, galactose, aminosugars, nucleotides, and arginine metabolism, the phosphotransferase system, and those related to the infectivity and quorum sensing system of *Staphylococcus*. The authors also stated that EA-Au-NPs exhibited significant activity against *E. faecium* (the MIC and MBC values both amounted to 3.12 μg·mL^−1^), *K. pneumoniae* (the MIC and MBC values equalled to 1.56 and 3.12 μg·mL^−1^, respectively), *A. baumannii* (the MIC and MBC values both were 3.12 μg·mL^−1^), *P. aeruginosa* (the MIC and MBC value were 1.56 μg·mL^−1^ and 3.12 μg·mL^−1^, respectively), and a strain of *E. coli* (depending on the strain, for Xen14 the MIC and MBC values were 0.78 and 1.56 μg·mL^−1^, while for DH5α they amounted to 0.39 and 0.78 μg·mL^−1^, respectively) [87]. Another study evaluated the properties of EA-loaded magnetic nanoparticles (Fe_3_O_4_-EA-NPs) against *E. coli* isolates. In the Kirby–Bauer test, the zone of inhibition value was in the range of 12–16 mm. In turn, the MIC values for these isolates were in the range of 0.19–1.56 mg·mL^−1^. Very promising results were obtained in the biofilm eradication test, where the eradication rate was in the range of 43–62%, using a nanoparticle concentration of 0.5xMIC. In a study with ethidium bromide (EtBr), Fe_3_O_4_-EA-NPs were shown to reduce efflux pump activity as early as the 1/8xMIC concentration. Particularly important is that the study proved that Fe_3_O_4_-EA-NPs exposure led to a decrease (by >40%) in the expression of key genes encoding the components of efflux pumps, namely, *acrB-1*, *acrB-2*, *tolC-1*, and *tolC-2* [88]. 

#### 2.5.2. In Vivo Assessment of Antimicrobial Activity of Ellagic Acid Nanoparticles (EA-NPs)

In vivo analysis of the antibacterial activity of the chitosan/zeine/gelatin film loaded with EA nanoparticles showed that the prepared film promoted the wound healing process [84].

Moreover, in an in vivo murine model of *A. baumannii*-induced sepsis among individuals in immunosuppression, the EA in the form of lipid NPs led to a significantly higher survival of laboratory animals. Liposomal therapy with the NPs at concentrations of 50 mg·kg^−1^ and 100 mg·kg^−1^ led to an increase in the survival rate to 30% and 60%, respectively. Furthermore, the therapy also resulted in a decrease in liver and kidney toxicity, as well as a reduction of inflammatory lung damage (decrease in IL-1β, IL-6, and TNF-α in broncho-alveolar fluid) [86].

Furthermore, the study of antimicrobial properties of Au-NPs modified with EA investigated in the in vivo Xen36-induced murine model of peritonitis provided highly insightful results; namely, the survival rate for the NPs group equalled 100% in comparison to 83% for the individuals treated with gentamicin [87].

#### 2.5.3. Antimicrobial Activity of Nanoparticles Based on Plant Extracts Containing Ellagic Acid

The antibacterial activity of silver nanoparticles loaded with aqueous extract of *Aerva lanata* (*Ae*E-Ag-NPs), in which ellagic acid was the most abundant of polyphenol compounds, was investigated in relation to clinical isolates of *S. aureus*. The MIC value oscillated at 15 μg·mL^−1^, and the MBC value was 20 μg·mL^−1^. In the Kirby–Bauer test, the measured zone of inhibition (ZOI) had a diameter of 21 mm, while for ciprofloxacin (CYP) used as a control group, the ZOI was 24 mm. The synthesised *Ae*E-Ag-NPs were also active against clinical isolates of *E. faecalis*. The diameter of the ZOI was 23 mm, while for the group treated with CYP, it was 29 mm. In the microdilution test, the MIC and MBC values amounted to 5 and 10 μg·mL^−1^, respectively. It was also found that *E. coli* clinical isolates were sensitive to silver nanoparticles loaded with *Aerva lanata* extract. Antimicrobial activity was determined both by the Kirby–Bauer assay (for the *Ae*E-Ag-NPs, the ZOI was 22 mm, while for the CYP, it amounted to 24 mm) and by microdilution (the MIC value was 5 μg·mL^−1^ and the MBC value was 10 μg·mL^−1^). Moreover, the *Ae*E-Ag-NPs also showed a strong activity against *P. aeruginosa* clinical isolates. The antibacterial activity was expressed through the prism of the MIC, and MBC values amounted to 5 and 10 μg·mL^−1^, respectively. In the Kirby–Bauer assay, the observed zone of inhibition was 21 mm, while for the control group (CYP), it was 24 mm [89]. Further studies on the antimicrobial activity of Ag-NPs synthesised using EA, derived from pomegranate (*Punica granatum*) extract against *S. aureus,* were conducted by Fernandes et al. [90]. The authors posited that these nanoparticles were characterised by high activity against tested bacteria, and the MIC value equalled 0.26 μg·mL^−1^ [90].

The utilisation of *P. granatum* leaf extract, containing ~43 μg·mL^−1^ of EA, for the production and antimicrobial activity of Ag-NPs can also be found in the work of Swilam et al. [91]. The tested concentrations ranged from 0.05 mg·100 µL^−1^ to 0.45 mg·100 µL^−1^. The Kirby–Bauer-assessed activity against tested pathogens showed a concentration-dependent effect against *S. aureus* with the ZOI at diameters of 11-17 mm. In the case of *E. coli*, the ZOI was estimated to be 12–18 mm, while for *P. aeruginosa,* the ZOI diameters were 11–16 mm [91]. The antibacterial efficiency of Ag-NPs obtained on the basis of *Terminalia chebula* seed extract, containing a high content of EA, against selected bacteria was demonstrated by the Kirby–Bauer analysis. The tested concentrations ranged from 25 to 75 μg·mL^−1^, and a concentration-dependent manner was observed. For *S. aureus,* the ZOI increased from 11.3 to 12.7 mm, while for *E. coli,* the ZOIs were between 12.3 and 13.4 mm [92]. Ekrikaya et al. [93] examined the antimicrobial properties of Ag-NPs based on extracts derived from blackberry (*Rubus fruticosus* L.; BBE) and raspberry (*Rubus idaeus* L.; RBE), which represent rich sources of ellagic acid. The exposure to the nanoparticles, at the concentration of 100 μg·mL^−1^, led to the eradication of almost 90% of *E. faecalis* cells in an in vitro study. The effectiveness was similar to that of classic disinfectants, for example, chlorhexidine or NaOCl [93].

Table 5 provides an overview of the antibacterial activity of ellagic acid-based nanoparticles.

There is a lack of studies regarding the impact of ellagic acid-based nanoparticles on *S. marcescens*. 

### 2.6. Proanthocyanidin (PAC)

#### 2.6.1. In Vitro Assessment of Antimicrobial Activity of Proanthocyanidin Nanoparticles (PAC-NPs)

**Figure 6:**
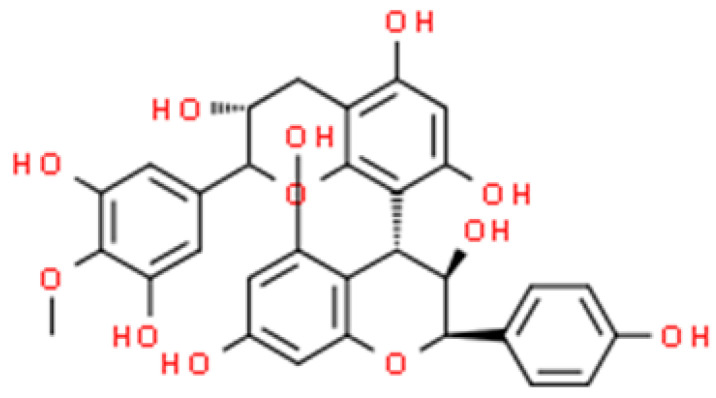
The structural formula of proanthocyanidin (ChemSpider database).

Alfaro-Viquez et al. [94] conducted a study on the properties of hybrid NPs of chitosan and PAC (for the chemical structure, see Figure 6) (PAC-CH-NPs) in the inhibition of invasive capacity and infectivity of extra-intestinal pathogenic *E. coli* strain 5011. The assessment of these NP properties was carried out in an in vitro experiment in a model of infection of Caco-2 cells. The exposure to PAC-CH-NPs, at the concentration of 100 μg·mL^−1^, led to an inhibition of invasiveness of the tested bacteria greater than 90%. Furthermore, SEM imaging provided evidence for the interaction of PAC-based NPs with the *E. coli* fimbriae P structure. It was noted that the fimbriae were characterised by extensive coating and cross-linking on multiple cells. This mechanism most likely prevented the ability of the pathogen to invade the intestinal epithelium [94]. In a similar study by Alfaro-Viquez et al. [95], SEM imaging showed that PAC-CH-NPs induced *E. coli* cell agglutination. The process involved disrupting the P-fimbriae and flattening the entire surface of the pathogen’s cell wall. This resulted in efficient precipitation of bacterial sediment and significantly reduced the invasiveness of *E. coli* against enterocytes. In vitro analysis of the effect of PAC-CH-NPs on the invasiveness of *E. coli* showed a decrease in the ability to infiltrate Caco-2 enterocytes by about 60–75% [95].

In the study by Shejawal et al. [96], the antibacterial properties of iron and gold NPs combined with PAC were investigated. The activity of these nanoparticles was assessed against, among others, *S. aureus* using the Kirby–Bauer test. However, no significant antimicrobial activity was demonstrated. The zone of inhibition for the Au-based NPs was 12 mm, and for the Fe-based NPs, it was 6 mm, while for the CYP-treated group, it was 38 mm. The negligible antibacterial properties of the PAC-containing NPs in combination with Au or Fe were also demonstrated against the *E. coli* strain. The ZOIs were 6 and 0 mm, respectively, while for the control group treated with CYP, the ZOI was 40 mm. It was also found that *P. aeruginosa* was not sensitive to PAC-AU-NPs or PAC-Fe-NPs. The observed values of ZOI oscillated within the limits of 7 and 6 mm, respectively, while for the CYP-treated group, it was 35 mm [96]. A study on proanthocyanidin-chitosan composite NPs loaded with gentamicin (G) in relation to different bacteria was conducted by Alfaro-Viquez et al. [97]. In the study of agglutinative abilities, the PAC-CH-G-NPs were highly effective, and a dose-dependent increase in agglutination in relation to the free PAC was observed. The proanthocyanidin itself did not show significant activity against *E. coli* (the MIC and MBC values were 1920 and >3840 μg·mL^−1^, respectively), while the application of PAC-CH-NPs with added gentamicin exhibited higher activity (both the MIC and MBC values were 37.5 μg·mL^−1^). The PAC-CH-NPs loaded with gentamicin also showed effectiveness in the agglutination test of *P. aeruginosa*. The concentration-dependent agglutination rate of cells was noted, and the most promising results were obtained with the PAC-CH ratio of 15:1 and 30:1. As in the case of *E. coli*, *P. aeruginosa* was not sensitive to the free PAC but was sensitive to the nanoparticles (the MIC and MBC values were 37 and 74 μg·mL^−1^). However, it was found that the antibiotic combined with the NPs was less effective than its free form (the MIC value increased from 2 to 4 μg·mL^−1^). Moreover, the properties of the PAC-CH-G-NPs were also evaluated against *S. aureus*. The promising effects in the tests regarding the induction of cell agglutination were achieved with the ratio of PAC and CH equal to 30:1. The SEM imaging showed higher agglutinative efficacy of bacterial cells after the application of the nanoparticles in comparison to the free PAC. Moreover, the effectiveness of the nanoparticles with the addition of gentamicin was also examined, and it was shown that the antibiotic possibly obtained a synergistic relationship in PAC + CH complexes in relation to all the bacteria tested. Interestingly, the evaluation of antimicrobial activity using microdilution showed that despite the low activity of PAC (the MIC and MBC values of 960 and >3840 μg·mL^−1^, respectively) and CH (the MIC and MBC values equal to 500 μg·mL^−1^) against *S. aureus*, the use of the NPs with gentamicin lowered the MIC value of the antibiotic from 2 μg·mL^−1^ to 1 μg·mL^−1^ (the MIC and MBC values for NPs were 9.3 and 18.5 μg·mL^−1^) [97]. 

Ding et al. [98] examined the activity of chitosan-based NPs loaded with PAC against *S. aureus*. Based on the microdilution test, the activity of the PAC-CH-NPs was assessed, and the MIC value was equal to 1.25 mg·mL^−1^, while for the positive control (levofloxacin), the MIC was <0.039 mg·mL^−1^. The SEM analysis revealed that exposure to the PAC-CH-NPs resulted in cell wall deformation and increased cell membrane permeability. It was also found that the PAC-CH-NPs were more active against *E. coli* than *S. aureus*; the MIC value was at the level of 0.625 mg·mL^−1^, while in the group with levofloxacin, the MIC value was <0.039 mg·mL^−1^. The test of susceptibility of *P. aeruginosa* to the PAC-CH-NPs revealed that the MIC value was 1.25 mg·mL^−1^, and it can be assumed that the PAC-CH-NPs have a comparable activity against *P. aeruginosa* as against *S. aureus* [98]. Wang et al. [99] evaluated PAC-coordinated ZnO and Ag NPs modified with PAC-coordinated ZnO activity in relation to, among others, *S. aureus*. The microdilution assay showed that the MIC value for both NP forms was equal to 0.15625 mg·mL^−1^. Particularly important is the fact that both forms of NPs in the Tryptic Soy Agar medium led to the ≥95% inhibition of the *S. aureus* cell growth. In an in vitro model of bone infection, the NPs showed several different mechanisms of antibacterial activity, namely the formation of ROS (ZnO and silver NPs) and antioxidant protective effects on body tissues (PAC). The PAC-coordinated ZnO and silver NPs-modified and PAC-coordinated ZnO described above also showed activity against *E. coli*. However, the MIC values were significantly higher than those observed for *S. aureus* and oscillated at values of 0.625 and 0.3125 mg·mL^−1^, respectively. Both treatments (in the TSA medium) led to an inhibition of growth for up to 95% of bacterial cells [99]. In Table 6, the antimicrobial activity of proanthocyanidin-based nanoparticles is shown.

#### 2.6.2. In Vivo Assessment of Antimicrobial Activity of Proanthocyanidin Nanoparticles (PAC-NPs)

There are no available in vivo studies in the literature regarding the antibacterial activity of PAC-NPs.

#### 2.6.3. Antimicrobial Activity of Nanoparticles Based on Plant Extracts Containing Proanthocyanidin

In the work of Araya-Sibaja et al. [100], a detailed study on the characterisation of a proanthocyanidin-enriched extract (PA-E) from *Uncaria tomentosa* leaves was presented. The authors synthesised two types of hybrid nanoparticles, a polymeric-lipid (F-1) and a protein-lipid (F-2), both loaded with PA-E. In vitro release, antioxidant activity via DPPH, and in vivo delayed-type hypersensitivity (DTH) reaction were evaluated. The results showed that PA-E enhanced its release rate and antioxidant activity when delivered in nanoparticle form, while in vivo, the nanoformulation increased immune stimulation, leading to augmented antigen-specific responses [100].

There is a lack of research on the effects of nanoparticles on *Enterococcus* spp., *K. pneumoniae*, *A. baumannii*, and *S. marcescens*, which underscores the importance of future studies to fill this gap in knowledge.

### 2.7. Sanguinarine (SG)

#### 2.7.1. In Vitro Assessment of Antimicrobial Activity of Sanguinarine (SG) Nanoparticles (SG-NPs)

**Figure 7:**
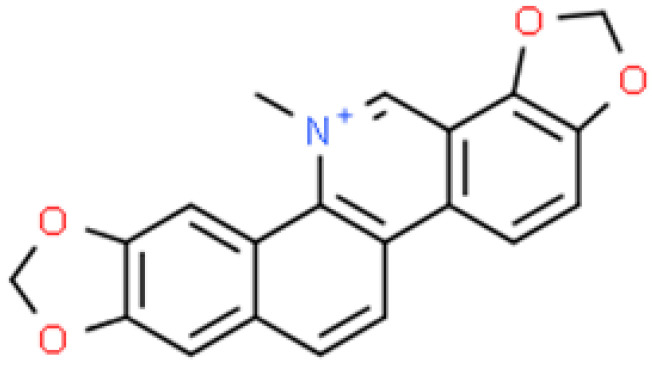
The structural formula of sanguinarine (ChemSpider database).

Only two studies investigated the impact of sanguinarine-based nanoparticles. In the first published article, the activity of p-sulfonatocalix[6]arene-functionalised silver nanoparticles with SG (the structural formula is shown in Figure 7) was evaluated against *S. aureus* and *E. coli*. The study of Gram-positive bacteria showed that the use of SG in the form of NPs (SG-NPs) led to a decrease in the MIC values for SG from 80 to 40 µM. It was also found that *E. coli* was sensitive to the treatment, and a decrease in the MIC values for SG from 20 to 10 µM was noted. Interestingly, the NPs showed a higher effectiveness in fighting *E. coli* than *S. aureus* [101]. In the second study, Andima et al. [33] evaluated the effectiveness of lactoferrin NPs loaded with SG with the addition of a classic antibiotic (imipenem or vancomycin). The study demonstrated clinically important interactions of the tested alkaloid with antibiotics. With regard to *S. aureus*, the combination of SG and imipenem was identified as additive activity, while SG and vancomycin as synergistic (reduction of the MIC value of SG from 12.5 to 2.0 μg·mL^−1^ and vancomycin from 1.25 to 0.156 μg·mL^−1^). The authors also evaluated the antibacterial properties of lactoferrin-SG-antibiotic-NPs against *S. aureus* in an in vitro assay. As expected, the combination of SG-imipenem/vancomycin-NPs effectively reduced the size of the *Staphylococcus* colony by 1–3 log units (the concentration of the NPs was 50 μg·mL^−1^) [33].

#### 2.7.2. In Vivo Assessment of Antimicrobial Activity of Sanguinarine (SG) Nanoparticles (SG-NPs)

Zhang et al. [102] evaluated antimicrobial activity of zwitterions-modified MXene quantum dot nanocarrier for SAN (SAN@AHEP@Ta4C3) in an in vivo model of wound infection in mice. The preparation was tested against both *E. coli* and *S. aureus*. The study explored the combined effects of the chemotherapeutic and photothermal properties of the nanocarriers, which were associated with improved wound healing and bacterial eradication. In an in vitro assessment, 85% growth inhibition was achieved for *E. coli* and approximately 90% for *S. aureus* after exposure to the nanocarriers. The suppression of bacterial proliferation in the wound site resulted in a reduction in the inflammatory response in the skin and a decrease in leukocyte activity [102].

#### 2.7.3. Antimicrobial Activity of Nanoparticles Based on Plant Extracts Containing Sanguinarine

There are no studies available in the literature regarding the antimicrobial activity of nanoparticles derived from plant extracts containing sanguinarine.

The antimicrobial activity of sanguinarine-based nanoparticles is presented in Table 7.

The effects of nanoparticles on *Enterococcus* spp., *K. pneumoniae*, *A. baumannii*, *S. marcescens*, and *P. aeruginosa* remain underexplored in the literature, highlighting a critical area for future research to investigate.

### 2.8. Safety of Using Nanoparticles and Current Regulations in Human and Veterinary Medicine

The use of nanoparticles in medicine, both topically and orally, has gained significant attention due to their potential to enhance the delivery and efficacy of therapeutic agents. However, their safety profile is a critical consideration, as their small size and unique properties can lead to interactions with biological systems that differ from bulk materials [103,104].

When applied topically, nanoparticles can penetrate the skin barrier, potentially leading to systemic exposure. Studies have shown that some nanoparticles, depending on their composition and surface properties, may cause skin irritation or even toxicity [105,106,107]. Oral administration presents additional risks, as nanoparticles can pass through the gastrointestinal tract and potentially accumulate in organs such as the liver, kidneys, and spleen [108,109]. Toxicological studies are essential to assess the long-term safety of nanoparticles, particularly regarding their accumulation in tissues, potential for inflammatory responses, and effects on cellular processes. In veterinary medicine, the safety concerns are similar, as animals may experience different responses to nanoparticles based on their size, species, and physiology [110]. Ensuring the safety of nanoparticles in veterinary medicine is particularly important when considering the use of nanoparticles in food animals, where residue accumulation may pose risks to both animal health and human consumers.

Currently, the regulation of nanoparticles in medicine varies across regions, but there is a growing consensus on the need for specific guidelines due to their unique characteristics. In the European Union, the use of nanomaterials in medical products is governed by the European Medicines Agency (EMA) and the European Commission’s Regulation on cosmetic products. These regulations require manufacturers to assess the safety of nanoparticles, including their physicochemical properties, potential for toxicity, and environmental impact [111]. In the United States, the Food and Drug Administration (FDA) has issued guidance on the use of nanotechnology in food, drugs, and cosmetics, requiring rigorous testing and labelling for products containing nanoparticles. Both regulatory bodies emphasise the need for pre-market evaluation and post-market surveillance to ensure safety [112]. In veterinary medicine, nanoparticles are also subject to regulatory oversight, though specific guidelines may be less established than for human medicine. Veterinary drug products containing nanoparticles must undergo similar safety assessments as human products, including toxicological studies and considerations for environmental impact [113,114]. The FDA and EMA also regulate veterinary medicines, and the use of nanoparticles in these products is increasingly scrutinised. Additional considerations include the potential for nanoparticles to enter the food chain through animal products, which necessitates careful monitoring of residue levels and safety.

Therefore, while nanoparticles hold great promise in medicine, including in veterinary applications, their safety must be carefully evaluated through extensive preclinical and clinical studies. Ongoing regulatory efforts aim to balance innovation with patient and animal safety, ensuring that nanoparticles used in medical and veterinary applications are both effective and safe.

## 3. Methodology

This review cites studies from the Scopus, Web of Science, PubMed, ScienceDirect, and Google Scholar databases, covering publications from 1999 to 2024. Titles and abstracts were screened, followed by a full-text review of selected articles. Relevant data were extracted and synthesised thematically. Only peer-reviewed articles in English were considered. Given the narrative nature of this review, no formal systematic methodology was applied, and potential selection bias is acknowledged. A total of 114 studies were referenced. The database search involved checking the following keywords in the topic and abstract of articles: ‘nanoparticles’, ‘berberine’, ‘catechin’, ‘chelerythrine’, ‘cinnamaldehyde’, ‘ellagic acid’, ‘proanthocyanidin’, ‘sanguinarine’, ‘*Staphylococcus*’, ‘*Enterococcus*’, ‘*Klebsiella*’, ‘*Acinetobacter*’, ‘*Escherichia*’, ‘*Serratia*’, and ‘*Pseudomonas*’. Table 1, Table 2, Table 3, Table 4, Table 5, Table 6 and Table 7 consist of a list of plant compounds and their mechanisms of antimicrobial activity discussed in the current study.

## 4. Conclusions

The use of active compounds combined with nanoparticles for bacterial infection treatment represents a promising strategy in modern medicine. Nanoparticles, due to their unique physicochemical properties, enhance the bioavailability and stability of antimicrobial agents, leading to more effective and targeted therapies. These nanomaterials can facilitate the controlled release of active substances, improving therapeutic outcomes and reducing the risk of side effects. Additionally, their ability to interact with bacterial cell membranes and biofilms contributes to overcoming common challenges such as antibiotic resistance. However, despite their potential, further research is needed to fully understand the long-term safety and toxicity of nanoparticle-based treatments, as well as to optimise their application in clinical settings. Overall, the combination of active compounds with nanotechnology offers a promising avenue for combating bacterial infections, but careful assessment and standardisation of these approaches are crucial for their successful integration into clinical practice.

## Figures and Tables

**Table 2:** The antibacterial mechanisms of catechin-based nanoparticles.

The Antibacterial Mechanisms	Reference
increased attachment and damage to bacterial cell membrane	[58]
ROS production	[44,47,48,52,56,57]
inhibited bacterial adhesion to bacterial cell walls	[59]
binding with proteins and DNA; affecting the respiratory chain; disrupting metabolism	[60]
disruption of bacterial biofilm formation	[57]

**Table 3:** The antibacterial mechanisms of chelerythrine-based nanoparticles.

The Antibacterial Mechanisms	Reference
DNA fragmentation, caspase accumulation, membrane depolarisation, exposure to phosphatidylserine, and chromosome condensation increased attachment and damage to bacterial cell membrane	[61]
ROS production	[63]

**Table 4:** The antibacterial mechanisms of cinnamaldehyde-based nanoparticles.

The Antibacterial Mechanisms	Reference
interfere with bacteria by causing aggregation or precipitation, disrupting their normal functions and inhibiting growth	[80]
decrease in the mobility of bacterial cells	[68]
increased permeability of cell membrane, inhibition of the ATP-synthase	[78]
malfunction of critical cellular structures, thickening of bacterial cell walls, premature cell division, disintegration of cytosol and cell lysis	[66]

**Table 5:** The antibacterial mechanisms of ellagic acid-based nanoparticles.

The Antibacterial Mechanisms	Reference
decreased expression of genes related to purine, galactose, aminosugars, nucleotides and arginine metabolism; the phosphotransferase system; inhibition of expression of genes associated with QS	[87]
decreased expression of key bacterial genes encoding the components of efflux pumps: *acrB-1*, *acrB-2*, *tolC-1*, *tolC-2*	[88]

**Table 6:** The antibacterial mechanisms of proanthocyanidin-based nanoparticles.

The Antibacterial Mechanisms	Reference
agglutination of bacterial cells	[97]
bacterial cell wall deformation and cell membrane increased permeability	[98]
agglutinative interaction with the bacterial fimbriae P structure	[94]

**Table 7:** The antibacterial mechanisms of sanguinarine-based nanoparticles.

The Antibacterial Mechanisms	Reference
potentiation of antibiotics activity (β-lactams and glycopeptides)	[33]
